# Evaluating Nursing Care Quality Through Inpatient Falls and Its Association With the Patient Experience: A Registry Study in Finnish Acute Care Settings

**DOI:** 10.1155/jonm/9949731

**Published:** 2026-05-19

**Authors:** Anu Nurmeksela, Asta Heikkilä, Santtu Mikkonen, Kristiina Junttila, Marja Kaunonen, Tiina Kortteisto, Jaana Peltokoski, Marita Ritmala, Susanne Salmela, Tarja Tervo-Heikkinen

**Affiliations:** ^1^ Department of Nursing Science, University of Eastern Finland, Kuopio, Finland, uef.fi; ^2^ Wellbeing Services County of Southwest Finland, Turku University Hospital, Turku, Finland, vsshp.fi; ^3^ Department of Nursing Science, University of Turku, Turku, Finland, utu.fi; ^4^ Department of Environmental and Biological Sciences, University of Eastern Finland, Kuopio, Finland, uef.fi; ^5^ Nursing Research Center, University Hospital, Helsinki, Finland, chru-strasbourg.fr; ^6^ University of Helsinki, Helsinki, Finland, helsinki.fi; ^7^ Faculty of Social Sciences, Tampere University, Tampere, Finland, uta.fi; ^8^ Wellbeing Services County of Pirkanmaa, Tampere University Hospital, Tampere, Finland, pshp.fi; ^9^ Wellbeing Services County of Central Finland, Jyväskylä, Finland; ^10^ Helsinki University Hospital, Helsinki, Finland, hus.fi; ^11^ Wellbeing Services County of Ostrobothnia, Vaasa, Finland; ^12^ Wellbeing Services County of North Savo, Kuopio University Hospital, Kuopio, Finland, psshp.fi

**Keywords:** inpatient falls, patient experience, quality of care, register study

## Abstract

**Background:**

The systematic collection and evaluation of evidence on the patient experience and the quality of care is crucial in advancing patient‐centred safe care. This study examined the association of the incidence of injurious inpatient falls with the patient experience of nursing care in Finnish hospitals.

**Aim(s):**

The aim was to determine how frequently inpatient falls occur, how patients evaluate their nursing care experience and whether these two factors are associated.

**Methods:**

A cross‐sectional registry study design was used. Data on inpatient falls, using hospital records and patient experience of nursing care, collected using the Nursing‐Sensitive Patient‐Reported Experience Measure (NsPREM), were collected in 2021 and 2022 from five university hospitals and four central hospitals in Finland. The data included a total of 4733 inpatient falls, of which 1899 were injurious, and 16,610 patient experience measures. Descriptive statistics were used to describe the incidence of inpatient falls and the patient experience, and linear mixed models were employed to examine the interaction between injurious inpatient falls and the patient experience. Analyses were conducted at a unit‐type group level.

**Results:**

Of the inpatient falls, 40% (*n* = 1899) caused injury and were statistically significantly associated with the patient experience measures, which are thus included in the linear mixed modelling. Overall, the patients experienced their care as excellent (with a mean greater than 4 on a five‐point scale) in every unit‐type group. Several subscales of NsPREM displayed associations with injurious inpatient falls. The patients were satisfied with nursing care on every subscale, particularly in terms of the courtesy and respect with which nurses treated them (mean: 4.83) and in terms of pain management (mean: 4.80). In linear mixed models, adjusting for injurious patient falls, the patients in psychiatric units reported that they were less satisfied with their care on every subscale compared with other unit‐type groups: for example, the patients’ experience of safety (Est. = −0.262, *p* < 0.001) and patient centredness (Est. = −0.399, *p* < 0.001) were significantly weaker in the psychiatric units.

**Conclusions:**

The findings show that continuous improvement requires ongoing monitoring and the evaluation of strategies to reduce inpatient falls and improve patient satisfaction, and there is a need for the ongoing training of nursing staff and targeted interventions for high‐risk patients. Hospital organisations must engage patients in their care, particularly in relation to fall prevention, to empower them and enhance their overall experience.

## 1. Introduction

The World Health Organization defines the *quality of care* as the degree to which health services increase the likelihood of reaching desired health outcomes for individuals and populations. It is based on evidence‐based professional competence and is critical to achieving universal health coverage. High‐quality health services must be effective, safe and people‐centred, delivering evidence‐based care that avoids harm and aligns with patients’ preferences, needs and values [1]. They must also be accessible and widely utilised. As users of health services, patients play a key role in assessing the quality of care [2]. Patients’ needs, expectations and values influence their evaluation of care quality, and their satisfaction increases when their experience of care involves effective communication, respect, dignity and emotional support [3]. Achieving high‐quality care outcomes requires uniform and comparable metrics and systematic arrangements for collecting and benchmarking outcomes. This would enable care quality to be continuously developed and improved. Care quality includes safety, of which patient falls are one indicator [4–6].

Nursing quality is essential in providing safe patient care and requires continuous monitoring [7]. In 2016, all five university hospitals in Finland started to collaborate on collecting and benchmarking nursing‐sensitive outcomes. Since then, several organisations have joined, and 18 of the 23 Wellbeing Services Counties are now members of the Finnish consortium for the national benchmarking of nursing‐sensitive outcomes (hereafter, *the consortium*). The consortium collects and benchmarks several nursing‐sensitive quality indicators, including the patient experience of nursing care and inpatient falls in Finnish social and healthcare organisations, fostering quality assurance and ensuring safe, effective and optimal care for patients and clients [8].

Patient‐centred care is well‐established in Finnish hospitals, and information about patient experience is routinely collected [9, 10]. Patients’ perceptions of their healthcare experiences have previously been assessed within their organisational contexts [11, 12]. This study collects data on patients’ experiences of the nursing care received in each unit of the studied hospitals, aiming to identify strengths and areas for improvement [10]. Hospitals have developed various models for improving patient‐centred care. For example, patient participation and involvement activities have been promoted, and quality‐improvement committees and hospital customer panels have been established [9]. Patient participation in safety interventions to reduce hospital falls has improved patient satisfaction [13].

Falls are a major global cause of death and disability, significantly contributing to years of life lost, disability and disability‐adjusted life years across all age groups [14]. Inpatient falls are the most common hospital adverse events [15]. Over a third of inpatient falls cause injuries, worsening clinical outcomes and increasing healthcare costs [6]. Injurious inpatient falls are internationally monitored and have significant consequences for patient care, often requiring additional resources and increasing healthcare costs [16]. They also contribute to a heightened fear of falling, causing psychological distress alongside physical harm and suffering [17]. However, most inpatient falls can be prevented through targeted nursing interventions with high‐risk patients [18]. Inpatient falls are associated with a complex array of individual, relational and environmental factors [19]. Hospitals that allocate increased budgets to evidence‐based practice experience fewer patient falls and injuries, underlining the role of evidence‐based approaches in enhancing care quality [20]. Quality‐accredited hospitals have fewer inpatient falls than other hospitals [21]. However, less is known about the patient experience relating to inpatient falls.

Recent evidence indicates that a patient safety culture and the patient experience are closely related concepts in hospital settings. A scoping review by Garrard et al. [22] found that both domains are shaped by organisational factors (such as communication, teamwork and leadership) and that a positive safety culture is frequently associated with more favourable patient‐reported experiences [23].

Previous studies have identified an association between hospital size and the patient experience: patients tend to be less satisfied and have worse experiences in hospitals with a larger number of beds. Further, patients at nonprofit hospitals have a more positive experience and are more likely to recommend the hospital than those at for‐profit hospitals. Patients are more satisfied with their care in specialised hospitals and those with academic status than in general hospitals [24]. Accredited hospitals have also achieved better patient experience scores and outcomes than nonaccredited hospitals [21]. In this article, we report on the quality of care in Finnish acute care settings, focusing on the patient experience and inpatient falls. The purpose was to determine how frequently inpatient falls occur, how patients evaluate their nursing care experience and whether these two factors are associated. In addition, associations between unit‐type groups and data collection years were investigated.

## 2. Materials and Methods

### 2.1. Design

A cross‐sectional registry design was adopted. The reporting of this study followed the Strengthening the Reporting of Observational Studies in Epidemiology checklist to ensure transparency, completeness and methodological rigour in presenting research findings.

### 2.2. Participants and Data Collection

Benchmarking data on inpatient falls and the patient experience of nursing care in 2021 and 2022 were collected from public healthcare organisations in Finland. In this study, data from five university hospitals and four central hospitals (the organisations which had produced both sets of data) were included. Data on inpatient falls (*n* = 4733) comprised monthly registry data that are continuously collected in the hospitals and were obtained either from a hospital’s data lake, based on nursing documentation, or through manual recording. Patient experience data (*n* = 16,610) were collected using paper or electronic surveys with adult inpatients (> 18 years old) across various medical specialties.

### 2.3. Instruments

#### 2.3.1. Inpatient Falls

The consortium has defined the benchmarking data for inpatient falls, which includes the number of patient days per month and the number of inpatient falls, categorised by injury level [1–5] (see Table 1). The injury classifications are based on the National Database of Nursing Quality Indicators framework [22]. Data on inpatient falls in hospitals were documented in an Excel‐based summary matrix.

**TABLE 1:** The injury levels of inpatient falls (*n*) during 2021‐2022 and fall incidence rates across 2,518,152 patient days.

Inpatient falls	*n*	Incidence rate^1^ ^)^
Falls with injury level 1^2^ ^)^ (*n*)	2834	1.13

Falls with injury level 2–5 (*n*) ‐ Injury level 2^3^ ^)^ (*n* = 1434) ‐ Injury level 3^4^ ^)^ *n* = 360) ‐ Injury level 4^5^ ^)^ (*n* = 90) ‐ Injury level 5^6^ ^)^ (*n* = 15)	1899	0.75
Inpatient falls total (*n*)	4733	1.88

^1)^Incidence rate: number of falls per 1000 patient days.

^2)^1 = None: no injury.

^3)^2 = Minor: minor injury, superficial injury.

^4)^3 = Moderate: moderate injury.

^5)^4 = Major: major injury, serious injury, significant injury.

^6)^5 = Death: fatality, injurious fall resulting in death [22].

#### 2.3.2. The NsPREM

The NsPREM was used to study the patients’ perceptions of their personal experience of nursing care. The NsPREM includes 22 items across nine subscales: care coordination (2 items); service recovery (2 items); safety (4 items); courtesy and respect (3 items); responsiveness (2 items); patient education (4 items); patient engagement/patient‐centred care (1 item); pain (2 items); and careful listening (2 items). The patients responded using a five‐point Likert scale (5 = *strongly agree*; 1 = *strongly disagree*) without any demographic questions. The patients also had the response option ‘It does not concern me’. In this study, we defined patient experience outcomes as *excellent* if the mean score was over 4.

The instrument was developed at Helsinki University Hospital in cooperation with the consortium. Item themes were developed based on the national healthcare client questionnaire and patient complaints received. Subscales were selected from the Magnet Hospital model [7]. Content validity was established through evaluations by nursing experts and patients. In a previous study, the Cronbach’s alpha for the total NsPREM was 0.960, with values of 0.75 for the safety subscale and 0.90 for the patient education subscale [25]. The low number of items under the other subscales made calculating the Cronbach’s alpha value irrelevant for them [26]. In this study, Cronbach’s alpha values were 0.86 for the total NsPREM, 0.84 for the safety subscale and 0.89 for the patient education subscale.

### 2.4. Ethical Approval

The study was conducted following the ethical principles of the Helsinki Declaration [27]. Permissions for the research were granted by the relevant research organisations in 2021. The study did not meet the criteria for prior ethical review under the guidelines of the Finnish National Board on Research Integrity (TENK). According to TENK, an ethics review is required in human sciences research only when specific conditions are met, such as intervention in a participant’s physical integrity, exposure to exceptionally strong stimuli, research involving minors without guardian consent, or circumstances in which the participant’s voluntariness may be compromised. None of these conditions were present in this study [28]. Data were handled in accordance with organisational data protection and information security procedures. The inpatient fall data and the NsPREM patient experience feedback used in this study are routinely collected organisational quality management data. Providing NsPREM feedback is voluntary customer feedback.

### 2.5. Statistical Analyses

In this study, we used the national benchmarking data, which is aggregated at a unit‐type level by nature, and identifications of exact units are removed. As an example, a specific code for the unit type is a ‘respiratory medical unit’. These unit‐type classifications have more than 20 unit types for adult inpatient care. Thus, to avoid excessively fragmented results, the two datasets were merged and aggregated further to the level of unit‐type groups (i.e., into groups of surgical, psychiatric, internal medicine, maternity and intensive/intermediate units).

Descriptive statistics—including frequencies, percentages, means and standard deviations—were used to summarise the data. Injurious inpatient falls classified with injury levels 2–5 were included in further analysis to ensure clinical relevance and a focus on events with direct implications for patient safety and care quality. To examine the association between injurious inpatient falls and the patient experience, we applied linear mixed models (LMMs) [29]. LMMs are an extension of traditional linear regression that account for both fixed and random effects, making them suitable for analysing hierarchical or clustered data. LMMs allow for correlation within clusters, in this case, accounting for variations between healthcare organisations. The models were adjusted for year and unit‐type group as fixed effects. To account for the hierarchical structure of the data, organisation ID was included as a random intercept term.

All statistical analyses were conducted using the mean values and rates of the unit‐type groups. Injurious inpatient fall data were expressed as incidence per 1000 patient days. For patient experience data, subscale means and an overall experience score were calculated. In LMM analysis, injurious inpatient fall rates and mean patient experience scores at the unit‐type group level were used. All analyses were conducted using IBM SPSS Statistics 29.0.2.0 (Armonk, NY: IBM Corp).

## 3. Results

### 3.1. Incidence of Inpatient Falls

Table 1 shows inpatient falls (*n* = 4733) according to their injury level [1–5], across a total of 2,518,152 patient days. Most of the falls (60%) resulted in level 1 injuries, suggesting no harm. Approximately 30% of the falls were classified as level 2, indicating minor harm, while 8% were level 3, representing moderate harm. Only a small proportion (about 2.2%) resulted in level 4 or 5 injuries, corresponding to major harm or death. Falls resulting in injuries at levels 2–5 occurred at a rate of 0.75 per 1000 patient days and were included in the further analysis presented in this report.

### 3.2. The Patient Experience

Overall, the patients rated their experience of nursing care highly, giving it a score between 4.2 and 4.7. However, the patient experience varied across the unit‐type groups, as measured on the NsPREM subscales. The patients were most satisfied in internal medicine and surgical units. The patients in psychiatric units were less satisfied on every subscale of the NsPREM (see Table 2).

**TABLE 2:** The mean values of patient experience subscales and total patient experience (scale: 1–5).

	**Mean**	**N**	**SD**	**Min–max**	**Mean**	**N**	**SD**	**Min–max**	**Mean**	**N**	**SD**	**Min–max**
	**Surgical units**				**Psychiatric units**				**Internal medicine units**			

Care coordination	4.73	214	0.41	1.00–5.00	4.15	95	0.45	2.00–5.00	4.75	270	0.28	2.83–5.00
Service recovery	4.59	211	0.44	1.00–5.00	3.96	93	0.52	2.00–5.00	4.63	262	0.36	3.00–5.00
Safety	4.40	214	0.35	1.25–5.00	3.68	96	0.58	1.00–5.00	4.40	271	0.39	3.00–5.00
Courtesy and respect	4.83	214	0.24	2.50–5.00	4.44	95	0.35	2.25–5.00	4.84	271	0.21	3.50–5.00
Responsiveness	4.78	214	0.28	2.00–5.00	4.27	95	0.37	2.25–5.00	4.79	271	0.25	3.33–5.00
Patient education	4.76	214	0.29	1.50–5.00	4.16	95	0.43	2.00–5.00	4.73	271	0.28	3.25–5.00
Pain	4.80	213	0.33	1.00–5.00	4.17	94	0.46	2.00–5.00	4.78	269	0.31	2.75–5.00
Careful listening	4.78	214	0.35	1.50–5.00	4.33	95	0.35	3.46–5.00	4.81	271	0.24	3.00–5.00
Patient engagement/patient‐centred care	4.73	213	0.43	1.00–5.00	4.21	95	0.43	2.00–5.00	4.73	269	0.28	3.00–5.00
Total patient experience	4.71	215	0.36	1.41–5.00	4.18	96	0.36	2.30–4.95	4.73	271	0.23	3.14–5.00

	**Maternity units**				**Intensive care and intermediate care units**				**All unit-type groups**			

Care coordination	4.64	92	0.51	1.00–5.00	4.64	54	0.48	2.00–5.00	4.65	725	0.44	1.00–5.00
Service recovery	4.50	91	0.61	1.00–5.00	4.54	50	0.44	3.20–5.00	4.51	707	0.50	1.00–5.00
Safety	4.41	92	0.47	1.00–5.00	4.29	54	0.60	2.00–5.00	4.30	727	0.50	1.00–5.00
Courtesy and respect	4.80	92	0.41	1.33–5.00	4.73	54	0.35	3.17–5.00	4.77	726	0.31	1.33–5.00
Responsiveness	4.71	92	0.33	2.50–5.00	4.72	54	0.39	3.00–5.00	4.70	726	0.34	2.00–5.00
Patient education	4.70	92	0.44	1.00–5.00	4.64	54	0.52	1.75–5.00	4.65	726	0.40	1.00–5.00
Pain	4.67	92	0.47	1.00–5.00	4.75	54	0.30	3.75–5.00	4.69	722	0.41	1.00–5.00
Careful listening	4.71	92	0.49	1.00–5.00	4.72	54	0.43	2.50–5.00	4.72	726	0.37	1.00–5.00
Patient engagement/patient‐centred care	4.71	92	0.51	1.00–5.00	4.64	53	0.50	2.00–5.00	4.65	722	0.43	1.00–5.00
Total patient experience	4.66	92	0.42	1.33–4.99	4.64	54	0.36	3.11–5.00	4.64	728	0.37	1.33–5.00

### 3.3. The Association of Inpatient Falls and Patient Experience

The LMMs revealed an association of injurious inpatient falls per 1000 patient days with seven subscales of patient experience. These subscales were care coordination, service recovery, safety, courtesy and respect, responsiveness, patient education, patient engagement/patient‐centred care and the total patient experience. The inpatient fall rate was negatively associated with several aspects of the patient experience, such as care coordination, safety, courtesy and patient‐centred care. For example, the safety assessment deteriorated significantly as the fall rate increased. Specifically, the higher the rate of inpatient falls, the lower the patients scored their experience of nursing care on these subscales (see Table 3).

**TABLE 3:** The association between the subscales of the patient experience, the total experience and inpatient falls (injury levels 2–5/1000 patient days) in five unit‐type groups in 2021 and 2022.

Falls/unit type/year	Care coordination			Service recovery			Safety			Courtesy and respect			Responsiveness		
	Est.	SE	*p*	Est.	SE	*p*	Est.	SE	*p*	Est.	SE	*p*	Est.	SE	*p*
Falls level 2–5/1000 patient days	−0.104	0.034	0.002	−0.058	0.028	0.037	−0.052	0.018	0.004	−0.052	0.018	0.004	−0.037	0.020	0.065
Intensive care and intermediate care unit (Reference)															
Surgical unit	0.155	0.085	0.085	0.222	0.076	0.008	0.247	0.048	0.001	0.183	0.048	0.001	0.156	0.059	0.009
Psychiatric unit	−0.452	0.092	< 0.001	−0.517	0.082	< 0.001	−0.262	0.052	< 0.001	−0.262	0.052	< 0.001	−0.425	0.064	< 0.001
Internal medicine unit	0.209	0.089	0.028	0.197	0.079	0.020	0.181	0.050	0.001	0.181	0.050	0.001	0.136	0.061	0.027
Maternity unit	0.060	0.098	0.548	0.065	0.086	0.461	0.120	0.056	0.044	0.120	0.056	0.044	0.037	0.068	0.585
2021 (2022 Reference)	0.081	0.039	0.040	0.028	0.032	0.371	0.039	0.020	0.057	0.039	0.020	0.057	0.017	0.023	0.452

**Falls/unit type/Year**	**Patient education**			**Patient engagement/patient-centred care**			**Pain**			**Careful listening**			**Total patient experience**		
	**Est.**	**SE**	**p**	**Est.**	**SE**	**p**	**Est.**	**SE**	***p***	**Est.**	**SE**	**p**	**Est.**	**SE**	**p**

Falls level 2–5/1000 patient days	−0.036	0.021	0.082	−0.057	0.028	0.042	0.005	0.019	0.782	0.008	0.018	0.660	−0.082	0.031	0.009
Intensive care and intermediate care unit (Reference)															
Surgical unit	0.208	0.055	0.001	0.196	0.069	0.008	0.112	0.070	0.112	0.110	0.055	0.055	0.140	0.081	0.099
Psychiatric unit	−0.390	0.060	< 0.001	−0.399	0.074	< 0.001	−0.616	0.076	< 0.001	−0.417	0.059	< 0.001	−0.396	0.087	< 0.001
Internal medicine unit	0.163	0.057	0.008	0.136	0.072	0.069	0.076	0.072	0.294	0.104	0.056	0.076	0.196	0.084	0.029
Maternity unit	0.130	0.064	0.053	0.152	0.078	0.066	−0.026	0.082	0.752	0.015	0.063	0.813	0.097	0.092	0.308
2021 (2022 Reference)	0.018	0.024	0.456	0.050	0.032	0.127	−0.006	0.021	0.772	0.011	0.020	0.597	0.054	0.036	0.132

*Note:* Est. = estimate, *p* = probability value.

Abbreviation: SE, standard error.

Table 3 shows the estimated values per unit‐type group and year compared with the reference unit‐type group (the intensive and intermediate care groups). The patients experienced lower satisfaction across all the subscales and in total in the psychiatric units than in the reference unit‐type group. For example, safety and patient‐centred care were significantly weaker. Further, the patients were more satisfied in terms of care coordination, safety, and courtesy and respect in 2021 than in 2022.

## 4. Discussion

This study examined the association between the incidence of inpatient falls and the patient experience of nursing care in Finnish hospitals in 2021 and 2022. The purpose was to determine how frequently inpatient falls occur, how patients evaluate their nursing care experience and whether these two factors are associated. The study provides a meaningful understanding of how adverse events relate to the patient experience and highlights the importance of targeted fall prevention strategies in nursing management. In this study, 1.88 inpatient falls occurred per 1000 patient days, with the largest proportion representing falls without injury. Overall, the patient experience of nursing care was rated as *excellent* across all unit‐type groups. Several of the NsPREM subscales demonstrated statistically relevant associations with injurious inpatient falls.

The incidence of injurious falls in our study, defined as an injury severity level greater than 1, was 0.75 per 1000 patient days. This rate is higher than the 0.4 per 1000 patient days reported in an earlier Finnish study [30]. The observed increase may reflect differences in patient populations, care environments or case‐mix severity. Although direct comparisons across studies should be interpreted cautiously, the findings indicate a need to further examine underlying contributors to injurious falls and to evaluate whether current fall prevention strategies and monitoring practices are sufficient within the studied care settings.

The results revealed that three‐quarters of the injurious inpatient falls resulted in minor harm (level 2). Inpatient falls leading to major harm or death (levels 4 and 5) were rare. These findings are consistent with previous research [16, 30]. Patients who experience falls in hospital may feel a loss of independence and autonomy, leading to disappointment, disempowerment and, in some cases, self‐blame for risk‐taking behaviour. Although falls increase patients’ reliance on nurses and shift their sense of control, they may also make patients more receptive to assistance and safety interventions [19]. Fall prevention interventions may improve patient knowledge and self‐efficacy and increase patient satisfaction [31]. Clear explanations of fall prevention strategies, supportive staff interactions and appropriate environmental modifications (such as handrails and low–low beds) can enhance patients’ awareness, confidence and willingness to follow safety guidance [32].

In this study, the patients experienced their care as *good* or *excellent* on every subscale, particularly those related to the courtesy and respect with which nurses treated them and pain management. Several previous studies have shown that patients are very satisfied with their care in Finnish hospitals [33, 34]. The finding that patients were very satisfied with the pain management conducted by nurses aligns with an earlier systematic review which showed that well‐managed pain was associated with higher overall patient satisfaction and more favourable patient interactions with clinical staff [35]. However, some previous studies have evaluated pain management as *poor* [33, 34, 36]. Effective pain management requires that nurses have received a diverse and systematic education [37], as nurse–patient communication and professional competence have been shown to relieve acute pain [36]. Barriers at the patient, professional and organisational levels must be addressed when developing and implementing evidence‐based practices [38]. Patient‐centred care is essential, as it is associated with improved pain relief outcomes [39].

In our study, the patients experienced less satisfaction with their care in psychiatric units on every subscale compared with other unit‐type groups: in particular, the patients’ experience of safety was low. Safety was also scored lowest of the subscales in other unit‐type groups. The primary data for this study were collected during the second wave of the COVID‐19 pandemic, which may have affected the patients’ experiences of security. The COVID‐19 pandemic challenged healthcare professionals in many ways as certain units were overloaded with patients and remote services were introduced during the lockdown. This caused a lot of uncertainty and fear among patients [40], and it is possible that psychiatric patients were particularly vulnerable to feeling insecure. During the pandemic, front‐line nurses were also under pressure, which manifested as mental health symptoms, stress and anxiety [41] and burnout [42]. There is strong evidence that burnout among nurses is associated with lower healthcare quality and safety and lower patient satisfaction [43].

The results showed that patients reported higher satisfaction on the subscales of care coordination, safety, and courtesy and respect in 2021 than in 2022, despite fewer inpatient falls during the latter year. Given that in 2021 the impact of the COVID‐19 pandemic was ongoing, this finding is notable [40].

We used LMMs to explore whether the incidence of injurious inpatient falls and the patients’ experience of their nursing care were associated. The results showed that when the inpatient fall rate was low, the patients experienced satisfaction with nursing care on several subscales including care coordination, service recovery, safety, courtesy and respect, responsiveness, patient education and patient engagement/patient‐centred care. Inpatient falls and their prevention are important indicators of patient safety culture and high‐quality care [44]. Effective communication and teamwork among healthcare professionals are key factors that link a patient safety culture with patient experience [23, 45]. Studies have shown that when nurses show courtesy and provide clarity, this positively influences patient satisfaction [46], with patients generally being more satisfied with being shown care and concern than they are with the information provided [47].

Our findings align with the recent study by Alabdaly et al. which reported that aspects of safety culture—including teamwork and communication—are commonly associated with a positive patient experience [23]. It is important that organisational values and efforts to improve the culture align with and are guided by patient‐centredness [48], as this also improves patients’ perception of safety [49]. Teamwork between healthcare professionals, patients and family members has proven to be effective in creating a climate of safety in hospitals. To capture different views of safety and better understand safety culture, particularly from the patient’s perspective, it is essential to create a trusting relationship that facilitates and encourages open communication and coordination between staff and patients [23]. Previous research has shown that a patient‐centred and effective healthcare culture improves patient outcomes, care experiences and patient satisfaction [48]. Patient‐centred care initiatives can also reduce the rate of falls [50].

Although this study was conducted in Finland, the findings on the association between injurious inpatient falls and the patient experience have broader relevance. The results may inform international nursing management practices by emphasising the value of patient‐centred care, proactive fall prevention strategies and the integration of patient experience data into quality and safety improvement efforts across diverse healthcare systems.

### 4.1. Limitations

The NsPREM is a relatively new instrument which has been developed within the Finnish healthcare context. This may limit its applicability to other settings. Although the NsPREM instrument has been validated with Cronbach’s alpha values between 0.861 and 0.90, its generalisability to international contexts requires further research.

Basically, the benchmarking data are collected on a unit‐type level. In some organisations, there may be multiple units with the same type. In this study, the data analysis was conducted on the level of unit‐type groups, including several similar unit types (such as surgical units). This limits the ability to identify within unit‐type group variation and may result in interpretations that reflect overall trends rather than detailed unit‐type dynamics.

As the register data was originally collected for benchmarking and developmental purposes, comprehensive background information was lacking. In addition, information on the units’ nurse staffing levels was not available in the benchmarking data and therefore could not be included as a covariate in the LMMs.

External factors, such as the COVID‐19 pandemic, may have influenced both the patient experience and the incidence and documentation of inpatient falls. External factors could have affected staffing levels, care continuity and patient perceptions of safety and responsiveness. Because the study was cross‐sectional, no causal conclusions can be drawn about the association between injurious inpatient falls and the patient experience. The observed associations may reflect shared underlying factors, including staffing levels or the overall safety culture. Thus, the findings should be interpreted as correlational rather than directional.

### 4.2. Implications for Nursing Management

This study provides valuable insights for nursing management by highlighting the importance of regularly evaluating fall prevention strategies and the patient experience to support continuous quality improvement. It emphasises the need for ongoing staff training, targeted interventions for high‐risk patients and active patient engagement in safety practices. These findings can inform leadership decisions and policy development aimed at enhancing nursing care and patient safety across diverse inpatient settings.

## 5. Conclusion

In this study, we explored the quality of care in Finnish acute care settings in terms of injurious inpatient falls and the patient experience. The patients in Finnish healthcare organisations reported high levels of satisfaction with nursing care, consistently rating it as *good* or *excellent*. There is a significant association between injurious inpatient falls and the overall patient experience: lower rates of falls are associated with higher patient satisfaction. Specific subareas—such as care coordination, safety and patient engagement—are crucial. However, the positive relationship between fewer falls and better patient experience does not hold for psychiatric units, indicating their unique challenges that demand specialised strategies. Healthcare organisations in Finland can continue to improve their already high quality of care and patient safety by focusing on the weaker areas identified in this study.

## Author Contributions

All the authors contributed to the conception and design of the study. Anu Nurmeksela, Kristiina Junttila, Asta Heikkilä and Tarja Tervo‐Heikkinen collaborated in compiling the data; Santtu Mikkonen was responsible for the data analysis, and Anu Nurmeksela, Kristiina Junttila, Asta Heikkilä and Tarja Tervo‐Heikkinen contributed to the interpretation of the findings. Anu Nurmeksela was responsible for writing the results section and drafting the manuscript. All the co‐authors (Asta Heikkilä, Santtu Mikkonen, Kristiina Junttila, Marja Kaunonen, Tiina Kortteisto, Jaana Peltokoski, Marita Ritmala, Susanne Salmela, Tarja Tervo‐Heikkinen) provided critical revisions and approved the final version of the manuscript.

## Funding

This work was supported by the Research Committee of the Wellbeing Services County of North Savo (Kuopio University Hospital) for the State Research Funding (Grant No: 1906/02.05.01/2023). Open access publishing facilitated by Ita‐Suomen yliopisto, as part of the Wiley ‐ FinELib agreement.

## Disclosure

All scientific content, analyses, and interpretations were produced by the authors. All the authors have read and agreed to the published version.

## Conflicts of Interest

The authors declare no conflicts of interest.

## Data Availability

The data that support the findings of this study are available on request from the corresponding author.

## References

[1] World Health Organization [WHO] , Quality of Care, https://www.who.int/health-topics/quality-of-care#tab=tab_1.

[2] Aiken L. H. , Sloane D. M. , Ball J. , Bruyneel L. , Rafferty A. M. , and Griffiths P. , Patient Satisfaction With Hospital Care and Nurses in England: An Observational Study, BMJ Open. (2018) 8, no. 1, 1–8, 10.1136/bmjopen-2017-019189, 2-s2.0-85040593951. PMC5781188 29326193

[3] Larson E. , Sharma J. , Bohren M. A. , and Tunçalp Ö. , When the Patient is the Expert: Measuring Patient Experience and Satisfaction With Care, Bulletin of the World Health Organization. (2019) 97, no. 8, 563–569, 10.2471/blt.18.225201, 2-s2.0-85070777817. 31384074 PMC6653815

[4] Aiken L. H. , Sloane D. M. , Bruyneel L. et al., Nurse Staffing and Education and Hospital Mortality in Nine European Countries: A Retrospective Observational Study, Lancet.(2014) 383, no. 9931, 1824–1830, 10.1016/s0140-6736(13)62631-8, 2-s2.0-84901197368. 24581683 PMC4035380

[5] Ministry of Social Affairs and Health , Client and Patient Safety, https://stm.fi/en/client-and-patient-safety.

[6] World Health Organization [WHO] , Patient safety, 2023, https://www.who.int/news-room/fact-sheets/detail/patient-safety.

[7] American Nurses Credentialing Center [ANCC] , About Magnet, https://www.nursingworld.org/organizational-programs/magnet/about-magnet/.

[8] HoiVerKe , The Finnish Consortium for the National Benchmarking of Nursing-Sensitive Outcomes, https://hoiverke.fi/en/.

[9] Nurmeksela A. , Tervo-Heikkinen T. , Junttila K. et al., Excellent Nursing Leadership Towards Magnet Culture Among Nurse Leaders: An Interview Study, Journal of Advanced Nursing. (2024) 00, no. 8, 1–13, 10.1111/jan.16662. PMC12271652 39722535

[10] HoiVerKe , Patient experience, The Finnish Consortium for the National Benchmarking of Nursing-Sensitive Outcomes. (2024) https://hoiverke.fi/en/nursing-sensitive-quality-of-care/patient-experience/.

[11] Casaca P. , Schäfer W. , Nunes A. B. , and Sousa P. , Using Patient-Reported Outcome Measures and Patient-Reported Experience Measures to Elevate the Quality of Healthcare, International Journal for Quality in Health Care. (2023) 35, no. 4, 10.1093/intqhc/mzad098. PMC10750971 38113907

[12] Bull C. , Teede H. , Watson D. , and Callander E. J. , Selecting and Implementing Patient-Reported Outcome and Experience Measures to Assess Health System Performance, JAMA Health Forum. (2022) 3, no. 4, 10.1001/jamahealthforum.2022.0326. 36218960

[13] Weber K. , Knueppel Lauener S. , Deschodt M. , Grossmann F. , and Schwendimann R. , Patient Involvement in Fall Prevention in an Acute Care Hospital: A Survey Study, International Journal of Nursing Science. (2024) 11. 10.1016/j.ijnss.2024.08.012 PMC11650666 39698139

[14] James S. L. , Lucchesi L. R. , Bisignano C. et al., The Global Burden of Falls: Global, Regional and National Estimates of Morbidity and Mortality From the Global Burden of Disease Study 2017, Injury Prevention. (2020) 26, no. Suppl 2, I3–I11, 10.1136/injuryprev-2019-043286. 31941758 PMC7571347

[15] Trinh L. T. T. , Assareh H. , Wood M. , Addison-Wilson C. , and Sathiyaseelan Y. , Falls in Hospital Causing Injury, Journal for Healthcare Quality. (2020) 42, no. 1, 1–11, 10.1097/jhq.0000000000000179. 30649003

[16] Dykes P. C. , Curtin-Bowen M. , Lipsitz S. et al., Cost of Inpatient Falls and Cost-Benefit Analysis of Implementation of an Evidence-Based Fall Prevention Program, JAMA Health Forum. (2023) 4, no. 1, 10.1001/jamahealthforum.2022.5125. PMC9860521 36662505

[17] Lee D. and Tak S. H. , A Concept Analysis of Fear of Falling in Older Adults: Insights From Qualitative Research Studies, BMC Geriatrics. (2023) 23, no. 1, 1–11, 10.1186/s12877-023-04364-5. 37821830 PMC10568775

[18] Montero-Odasso M. , Velde N. V. D. , Martin F. C. et al., World Guidelines for Falls Prevention and Management for Older Adults: A Global Initiative, Age and Ageing. (2022) 51, 1–36. 10.1093/ageing/afac205 PMC9523684 36178003

[19] Jarden R. J. , Cherry K. , Sparham E. et al., Inpatients’ Experiences of Falls: A Qualitative Meta-Synthesis, Journal of Advanced Nursing. (2024) 81, no. 1, 4–19, 10.1111/jan.16244. 38808473 PMC11638511

[20] Melnyk B. M. , Hsieh A. P. , Messinger J. , Thomas B. , Connor L. , and Gallagher-Ford L. , Budgetary Investment in Evidence-Based Practice by Chief Nurses and Stronger EBP Cultures are Associated With Less Turnover and Better Patient Outcomes, Worldviews on Evidence-Based Nursing. (2023) 20, no. 2, 162–171, 10.1111/wvn.12645. 37042488

[21] Connor L. , Beckett C. , Zadvinskis I. et al., The Association Between Magnet Recognition and Patient Outcomes: A Scoping Review, Journal of Nursing Administration. (2023) 53, no. 10, 500–507, 10.1097/nna.0000000000001325. 37695278

[22] Garrard L. , Boyle D. K. , Simon M. , Dunton N. , and Gajewski B. , Reliability and Validity of the NDNQI Injury Falls Measure, Western Journal of Nursing Research. (2016) 38, no. 1, 111–128, 10.1177/0193945914542851, 2-s2.0-84949786117. 25023824

[23] Alabdaly A. , Hinchcliff R. , Debono D. , and Hor S. Y. , Relationship Between Patient Safety Culture and Patient Experience in Hospital Settings: A Scoping Review, BMC Health Services Research. (2024) 24, no. 1, 1–10, 10.1186/s12913-024-11329-w. 39113045 PMC11308681

[24] Friedel A. L. , Siegel S. , Kirstein C. F. et al., Measuring Patient Experience and Patient Satisfaction—How are we Doing it and Why Does it Matter? A Comparison of European and U.S. American Approaches, Healthc.(2023) 11, no. 6, 1–15, 10.3390/healthcare11060797. PMC10048416 36981454

[25] Löfqvist C. and Ritmala-Castrén M. , Nursing Feedback Survey–Psychometric Properties of the Measurement [Hoitotyön Palautekysely–Mittarin Ominaisuudet], XVI National Nursing Conferences 309-1102020 Conference proceedings. (2020) Springer.

[26] Eisinga R. , Grotenhuis M. Te , and Pelzer B. , The Reliability of a Two-Item Scale: Pearson, Cronbach, or Spearman-Brown?, International Journal of Public Health. (2013) 58, no. 4, 637–642, 10.1007/s00038-012-0416-3, 2-s2.0-84880919385. 23089674

[27] World Medical Association , WMA declaration of Helsinki – ethical principles for medical research involving human subjects, World Medical Association. (2022) 310. 10.1001/jama.2013.281053 24141714

[28] Finnish National Board on Research Integrity [Tenk] , The Finnish Code of Conduct for Research Integrity and Procedures for Handling Alleged Violations of Research Integrity in Finland [Internet], Finnish National Board on Research Integrity. (2023) 1–32, https://tenk.fi/sites/default/files/2023-11/RI_Guidelines_2023.pdf.

[29] Brown H. and Prescott R. , Applied Mixed Models in Medicine, 2015, 3rd edition, John Wilet & Sons, Ltd.

[30] Heikkilä A. , Lehtonen L. , and Junttila K. , Fall Rates by Specialties and Risk Factors for Falls in Acute Hospital: A Retrospective Study, Journal of Clinical Nursing. (2023) 32, no. 15–16, 4868–4877, 10.1111/jocn.16594. 36478598

[31] Capezuti E. , Tan A. , Talatala R. A. et al., Interventions to Prevent Falls and Injuries in Inpatient Oncology Units: A Scoping Review, Journal of Nursing Care Quality. (2025) 40, no. 3, 209–216, 10.1097/ncq.0000000000000849. 40146975

[32] Yasan C. , Rohlje B. , Cork J. , Cooper-Blair C. , and Hayes A. , Systematic Review of Patients’ Perceptions of the Contributing Factors that Led to Falling in Acute Care Hospitals During Their Hospitalisation, Nursing Open. (2025) 12, no. 5, 10.1002/nop2.70240. PMC12096458 40401919

[33] Nurmeksela A. , Kulmala M. , and Kvist T. , Patient Satisfaction–Results of Cluster Analysis of Finnish Patients, BMC Health Services Research. (2023) 23, no. 1, 1–8, 10.1186/s12913-023-09625-y. 37312180 PMC10265807

[34] Kvist T. , Voutilainen A. , Mäntynen R. , and Vehviläinen-Julkunen K. , The Relationship Between Patients’ Perceptions of Care Quality and Three Factors: Nursing Staff Job Satisfaction, Organizational Characteristics and Patient Age, BMC Health Services Research. (2014) 14, no. 1, 1–10, 10.1186/1472-6963-14-466, 2-s2.0-84928788697. 25326852 PMC4283083

[35] Mazurenko O. , Collum T. , Ferdinand A. , and Menachemi N. , Predictors of Hospital Patient Satisfaction as Measured by HCAHPS: A Systematic Review, Journal of Healthcare Management. (2017) 62, no. 4, 272–283, 10.1097/jhm-d-15-00050, 2-s2.0-85024408362. 28683051

[36] Hämäläinen J. , Tarja K. , and Kankkunen P. , Exploratory Study of Patient Perceptions of Pain Management in Emergency Department, International Journal of Caring Sciences. (2021) 13, no. 3, 1547–1557.

[37] Grommi S. , Vaajoki A. , Voutilainen A. , and Kankkunen P. , Effect of Pain Education Interventions on Registered Nurses’ Pain Management: A Systematic Review and Meta-Analysis, Pain Management Nursing [Internet]. (2023) 24, no. 4, 456–468, 10.1016/j.pmn.2023.03.004. 37032260

[38] Liu J. , Li X. , Tan Y. , Hu M. , Fang Y. , and Wang J. L. , Barriers for Nurses Providing Cancer Pain Management: A Qualitative Systematic Review, Oncology Nursing Forum. (2023) 50, no. 3, 348–360, 10.1188/23.ONF.348-360. 37155977

[39] Jakobsson S. , Ringström G. , Andersson E. et al., Patient Safety Before and After Implementing Person-Centred Inpatient Care—A Quasi-Experimental Study, Journal of Clinical Nursing. (2020) 29, no. 3–4, 602–612, 10.1111/jocn.15120. 31769572

[40] Çal A. , Kabataş Yıldız M. , and Aydın Avci İ. , The Effect of Health-Related Media Messages on Fear and Uncertainty About the COVID-19 Pandemic, Disaster Medicine and Public Health Preparedness. (2024) 18, 10.1017/dmp.2024.124. 39291315

[41] Tao R. , Wang S. , Lu Q. et al., Interconnected Mental Health Symptoms: Network Analysis of Depression, Anxiety, Stress, and Burnout Among Psychiatric Nurses in the Context of the COVID-19 Pandemic, Frontiers in Psychiatry. (2024) 15, no. October, 1–10, 10.3389/fpsyt.2024.1485726. PMC11550990 39529901

[42] Galanis P. , Vraka I. , Fragkou D. , Bilali A. , and Kaitelidou D. , Nurses’ Burnout and Associated Risk Factors During the COVID-19 Pandemic: A Systematic Review and Meta-Analysis, Journal of Advanced Nursing. (2021) 77, no. 8, 3286–3302, 10.1111/jan.14839. 33764561 PMC8250618

[43] Li L. Z. , Yang P. , Singer S. J. , Pfeffer J. , Mathur M. B. , and Shanafelt T. , Nurse Burnout and Patient Safety, Satisfaction, and Quality of Care: A Systematic Review and Meta-Analysis, JAMA Network Open. (2024) 7, no. 11, 10.1001/jamanetworkopen.2024.43059. PMC11539016 39499515

[44] Oner B. , Zengul F. D. , Oner N. , Ivankova N. V. , Karadag A. , and Patrician P. A. , Nursing-Sensitive Indicators for Nursing Care: A Systematic Review (1997–2017), Nursing Open. (2021) 8, no. 3, 1005–1022, 10.1002/nop2.654. 34482649 PMC8046086

[45] Mazurenko O. , Richter J. , Kazley A. S. , and Ford E. , Examination of the Relationship Between Management and Clinician Perception of Patient Safety Climate and Patient Satisfaction, Health Care Management Review. (2019) 44, no. 1, 79–89, 10.1097/hmr.0000000000000156, 2-s2.0-85057524483. 28445323

[46] Alibrandi A. , Gitto L. , Limosani M. , and Mustica P. F. , Patient Satisfaction and Quality of Hospital Care, Evaluation and Program Planning. (2023) 97, no. September 2022, 10.1016/j.evalprogplan.2023.102251. 36806007

[47] Karaca A. and Durna Z. , Patient Satisfaction With the Quality of Nursing Care, Nursing Open. (2019) 6, no. 2, 535–545, 10.1002/nop2.237, 2-s2.0-85062976632. 30918704 PMC6419107

[48] Abid M. H. , Kumah A. , Newera A. , and Hafez P. , Patient-Centered Healthcare: From Patient Experience to Human Experience, Global Journal on Quality and Safety in Healthcare. (2024) 7, no. 4, 144–148, 10.36401/jqsh-24-x2. 39534234 PMC11554389

[49] Choi N. , Kim J. , and Kim H. , The Influence of Patient-Centeredness on Patient Safety Perception Among Inpatients, PLoS One. (2021) 16, no. 2 Febuary, 1–14, 10.1371/journal.pone.0246928. PMC7880440 33577622

[50] Rossiter C. , Levett-Jones T. , and Pich J. , The Impact of Person-Centred Care on Patient Safety: An Umbrella Review of Systematic Reviews, International Journal of Nursing Studies. (2020) 109, 10.1016/j.ijnurstu.2020.103658. 32593882

